# Ordered Lipid Domains Assemble via Concerted Recruitment of Constituents from Both Membrane Leaflets

**DOI:** 10.1103/PhysRevLett.124.108102

**Published:** 2020-03-13

**Authors:** Ali Saitov, Sergey A. Akimov, Timur R. Galimzyanov, Toma Glasnov, Peter Pohl

**Affiliations:** 1 Institute of Biophysics, Johannes Kepler University Linz, Gruberstraße 40, Linz 4020, Austria; 2 A.N. Frumkin Institute of Physical Chemistry and Electrochemistry, Russian Academy of Sciences, 31/5 Leninskiy prospekt, Moscow 119071, Russia; 3 Institute of Chemistry, University of Graz, Heinrichstr. 28, 8010 Graz, Austria

## Abstract

Lipid rafts serve as anchoring platforms for membrane proteins. Thus far they escaped direct observation by light microscopy due to their small size. Here we used differently colored dyes as reporters for the registration of both ordered and disordered lipids from the two leaves of a freestanding bilayer. Photoswitchable lipids dissolved or reformed the domains. Measurements of domain mobility indicated the presence of 120 nm wide ordered and 40 nm wide disordered domains. These sizes are in line with the predicted roles of line tension and membrane undulation as driving forces for alignment.

Cell plasma membranes often display lateral inhomogeneities [1]. Such component organization into nanodomains is thought to be required for protein functioning, i.e., for the recruitment of diverse lipid and proteinaceous interaction partners [2]. Domains between 10 and 200 nm in diameter are called rafts if they are rich in sphingomyelin and cholesterol [3]. Rafts in biological membranes are distinct from detergent resistant membranes [4]. Their intrinsic permeability to small molecules is reduced [5]. Much effort has been devoted to uncovering the role of rafts in cellular processes like exo- and endocytosis [6], signaling [7], apoptosis [8], viral infection [9], and immune defense [10]. Raft lipids are more than just a passive platform for functional proteins [11]: for example, they may act as scaffold structures for proteins involved in apoptosis [12].

Because of their small size, rafts are below the diffraction limit of light microscopy. Consequently, optical observations were thus far limited to model membranes, where domains reach larger sizes [13]. Two major classes are distinguished: liquid disordered domains (LDDs) and liquid ordered domains (LODs). Like rafts, LODs are enriched in saturated lipids and cholesterol [14,15]. Unsaturated lipids preferentially partition into LDDs. LODs appear thicker than LDDs. The resulting line tension γ at phase border forces LODs to adopt a circular shape in unsupported bilayers [14] that is quickly restored after perturbation [15].

Micrometer sized LODs from the two monolayers of an unsupported lipid bilayer appear to be always in register [16]. Conceivably, the same holds for (*α*) nanometer sized LODs or (*β*) rafts in a plasma membrane. However, proof for their registration is extremely scarce. There are four lines of support for the notion: (i) AFM experiments, (ii) theoretical considerations, (iii) molecular dynamics simulations, and (iv) simulation aided time resolved fluorescence resonance energy transfer experiments:

(i) The AFM experiments were carried out on supported lipid bilayers [17]. Yet the presences of both a solid support and a thin layer of water between the bilayer and the support preclude the unequivocal assertion that the thicker LODs [18] always span the membrane. First, the thickness of the confined water layer may vary, as it is determined by the balance between van der Waals attraction, hydration forces, and electrostatic interactions [19]. Second, LODs in the monolayer adjacent to the support appear to be immobile [20]. Both the altered mobility and support-bilayer interactions may affect registration.
(ii) Domain registration reduces *γ* along the LOD’s rim, thereby minimizing the total energy stored in the system [21]. The gain in energy is sufficient to support registration of 10 nm wide LODs. Yet an opposing theory claims that only forces that are proportional to the domain area may be of relevance [22]. Yet, coupling at the membrane midplane is too weak to drive nanodomain registration.
(iii) Cholesterol’s preference for saturated tails drives phase separation in ternary lipid mixtures [23]. Coarse-grained molecular dynamics simulations show 15 nm large bilayer spanning LODs, whereas the unsaturated lipids segregate into LDDs. Yet another set of coarse grain simulations revealed that domain coalescence in compositionally symmetric bilayers may result in phase asymmetry (domain antiregistration) between the two leaflets [24].
(iv) Exploiting fluorescence lifetime imaging of Förster resonance energy transfer in combination with Monte Carlo simulations suggested nanodomain registration in giant unilamellar vesicles [25]. These domains appear to be fluid and disordered [26]. Thus, registration of ordered, raftlike domains remains yet to be shown.

Here we used simple confocal imaging (LSM 510 META, Zeiss, Germany) to confirm the assembly of membrane spanning nanometer-sized LODs in a minimal (protein-free) system. Therefore, we formed solvent-depleted asymmetric planar membranes as previously described [27]. In brief, an aperture (~ 150 *μm* in diameter) in a Teflon diaphragm was lowered beneath lipid monolayers on top of the adjacent preheated aqueous solutions. The diaphragm was pretreated with 0.5% hexadecane in hexane. The monolayers differed in the lipid anchored dyes that they harbored: Atto565–DPPE, 1,2-dipalmitoyl-*sn*-glycero-3-phosphoethanolamine, or Atto633–PPE, 1-palmitoyl-2-hydroxy-*sn*-glycero-3-phosphoethanolamine (ATTO TEC GmbH, Siegen, Germany). Since both dyes preferentially partition into LDDs, the colocalizations of (i) dark membrane areas (LODs) from both leaflets with each other and (ii) bright membrane patches (LDDs) from the two leaflets with each other indicate domain registration (Fig. 1).

PhoDAG–1’s photoresponse [28] served to induce and dissolve LODs. This is due to the azobenzene switch in one of the acyl chains that may adopt *cis* or *trans* conformations (Fig. 2). It thus reliably allowed us to obtain a population of small domains that otherwise is scarcely observable in model membranes.

The photoinduced domains were able to change size. Predominantly, the domains grew due to collisions and merger with each other. However, the recruitment of membrane material also happened via simple lipid diffusion (Fig. 3, upper row). Vice versa, photoswitching of PhoDAG–1 into its *trans* state resulted in LDD dissolution or shrinkage, i.e., in a decrease of the apparent diameter, *d_a_*. The reduction of *d_a_* did not necessarily require the pinching-off of smaller domains (Fig. 3, lower row).

*d_a_* overestimates the actual diameter d by distance *δ* due to (i) domain movement during image acquisition (Fig. S1 [29]) and (ii) diffraction limitations (Fig. S2 [29]). A rough theoretical estimation [29] predicts (in nm) 340 < δ < 560 for LDDs in LODs and 440 < δ < 660 for LODs in LDDs, where δ does not depend on *d_a_* for *d_a_ <* 1.7 *μ*m.

Inferring d from the diffusion coefficient *D* of one kind of domain (either LOD or LDD) in the other phase appears feasible. For the analysis of domain diffusion we used the Mosaic/Particle Tracking 2D/3D plugin [30,31] of ImageJ (National Institute of Health, Bethesda, Maryland, USA). Only domain movement that was compatible with simple diffusion entered the analysis [29,32] (Fig. 4). We analyzed 29 dimmer LODs that (i) during the observation time did not change their size and (ii) diffused within bright LDDs. Their diffusion coefficients *D* depended on *d_a_* (Fig. S3 [29]). Repeating the same procedure for 31 size-invariant bright LDDs diffusing in dark LODs also revealed a dependence of *D* on *d_a_* (Fig. S3 [29]). The diffusion of both LDDs and LODs can be described by the Saffman-Delbrück relation if the parameter *ε = (dη_3D_/hη) <* 0.1 [33]. Considering bilayer thickness *h = 5* nm, membrane viscosity *η* = 0.5 Pa s (see below), water viscosity *η*_3D_ = 10^−3^ Pa s, and domain diameter d = 1 *μ*m, we find

(1) $$\varepsilon =\frac{d}{u}\beta =\frac{\left(d_{a}-\delta \right)}{h}\frac{\eta_{\text{3D}}}{\eta }=0.4,$$

where *β* = *η_3D_u*/(*hη*) and *u =* 1 *μ*m. In consequence we used the so-called generalized Saffman–Delbrück equation [34] that has recently been introduced for 10^−3^ < ε < 10^3^, i.e., for the diffusion of micrometer-sized domains [35]:

(2) $$D=A\frac{\ln\left(\frac{2}{\varepsilon }\right)-\gamma_{e}+\frac{4\varepsilon }{\pi }-\frac{\varepsilon^{2}}{2}\ln\left(\frac{2}{\varepsilon }\right)}{1-\frac{\varepsilon^{2}}{\pi }\ln\left(\frac{2}{\varepsilon }\right)+\frac{v\varepsilon^{p}}{1+w\varepsilon^{q}}},$$

where *A = k_B_T/(4πhη), γ_e_ =* 0.5772, *p =* 2.74819, *q =* 0.61465, *v =* 0.73761, and *w =* 0.52119.

We obtained the parameters A, *β*, and *δ* by fitting Eq. (2) to the experimentally observed dependencies of *D* on *d_a_* (Fig. S3 in the Supplemental Material [29]); Table I). Using the fit parameter *δ* we replotted *D* as a function of *d* (Fig. 5). *d* of the smallest LDD and LOD amounted to 40 ± 18 and 120 ± 60 nm, respectively.

We treated *A* and *β* as independent parameters to improve the quality of the fit. In theory they are linked via *βk_B_T/*(4*πuA*) = *η*_3D_. The accordingly calculated *η*_3D_ values did not significantly differ for LDDs and LODs. Yet the error was comparatively large. This prompted us to validate the parameters *A* and *β* by (i) predicting single lipid diffusion from the fit and (ii) measuring *D* of labeled lipids in LDDs and LODs by fluorescence correlation spectroscopy. The respective experimental values of 7.8 and 0.9 *μ*m^2^/s (Fig. 6) agree reasonably well with the ones extrapolated to diffusing entities [Fig. 5, Eq. (2)] that have the size of a single fluorescently labeled lipid (*d* = 0.9 nm): 6.2 and 1.6 *μ*m^2^/s, respectively. This calculation neglects the height differences between a lipid and a domain, because for lipids that span one or two leaflets differs D by only about 30% [36].

Even the smallest LODs and LDDs span the whole bilayer as indicated by the fluorescence intensity of the dyes in the two monolayers (Fig. 1), i.e., domains as small as 40–120 nm span the bilayer. In other words, both LODs and LDDs from the two membrane leaflets are in register starting from miniature sizes. Thus far, small domains evaded optical observation in nonsupported model systems [37], but appeared to be detectable by NMR [37,38]. Nanometer sized membrane domains were reported to exist in plasma membranes [39], yet registration of LODs in living cells was only postulated to happen. Experimental evidence has not yet been obtained, although asymmetric lipid composition does not preclude coupling of the two membrane leaflets [40]. Such elusiveness of rafts in cell membranes has called their mere existence into question [41].

We introduced a new approach for observing LODs by conventional laser scanning fluorescence microscopy. It is based on the use of the correction parameter that accounts for both the limited scanning speed and aberrations due to diffraction limitations. The validity of the generalized Saffmann-Delbruck diffusion equation for diffusing entities of diameters from *d* ~ 0.9 nm to *d* ~ 20 *μ*m was the only major supposition made to introduce *δ* The assumed invariance of *δ* on domain size was experimentally confirmed by extrapolating from domain diffusion to the mobility of single lipids (Fig. 6).

Additional support for the approach comes from a rough assessment of membrane viscosity data. Using Eq. (2) and the parameters listed in Table I, we obtain the viscosities of both the LOD phase, *η_o_* = 0.458 ± 0.092 Pas and the LDD phase, *η_d_* = 0.108 ± 0.036 Pas. These values correspond to *η_o_h=* (2.29 ± 0.46) × 10^−9^ and *η_d_h* = (0.43 ± 0.14) × 10^−9^ Ns/m, respectively. They agree well with published data (3.3 ± 1.1) × 10^−9^ [42] and ≈0.5 x 10^−9^ Ns/m [34].

An important technical advancement that allowed us to optically observe nanometer-sized domains is the use of photoswitchable lipids. They (i) triggered both domain dissolution and domain induction, and (ii) altered *γ* at the domain border in a way that stabilized domain size over an extended observation period. This is important because *γ* acts as the major driving force for registration of nanometer sized domains [21,43]. The registration of LODs (LDDs) from the two leaflets minimizes the energy *w* stored in the rim of every LOD (LDD) [21,43]. We used the following parameters to calculate *w*: LOD’s hydrophobic thickness per monolayer *h_R_ =* 1.8 nm, LDD’s hydrophobic thickness per monolayer *h_s_ =* 1.3 nm, splay modulus of the LOD monolayer *B_R_ =* 20 *k_B_T*, splay modulus of LDD monolayer *B_S_ =* 10 *k_B_T*, and lateral compression-stretching modulus *K_A_ =* 120 mN/m (per monolayer). The spontaneous curvatures *J_R_* and *J_S_* of coexisting phases were taken as indicated in Table II. Upon *cis-trans* photoswitching, the PhoDAG–1 molecular geometry (i.e., effective spontaneous curvature) is expected to change substantially from slightly conical in the *trans* state to strongly conical in the *cis* state. Since *γ* strongly depends on curvatures *J_R_* and *J_S_* [44], the photoswitching is expected to alter the line tension. We calculate *w* per unit length of the boundary as Δ*γ_cis_=* 0.2 *k_B_T/nm* (for cis-PhoDAG–1) and Δ*γ*_trans_ = 0.07 *k_B_T*/nm (for trans–PhoDAG–1) [21,43]. The specific energy gain *w*_area_ upon registration of ordered domains driven by membrane shape undulations [45] is given by the following Eq. [46]:

$$w_{\text{area}}=\frac{k_{B}T}{4a^{2}}\text{ln}\left[\frac{{\left(B_{S}+B_{R}\right)}^{2}}{4B_{S}B_{R}}\right],$$

where *a* is the ultraviolet cutoff parameter of the undulations, which is of the order of 1 nm. We find *w*_area_ = 0.013 *kBT/nm^2^* for splay moduli *B_R_ = 2B_S_*.

For domains of *d* = 40 nm, *w_cis_* = 24.33 *k_B_T*, w_trans_ = 8.33 *k_B_T* (for cis–and trans–PhoDAG–1, respectively), and *W*_area_ = 15.56 *k_B_T*. Thus, registration of these small domains is mainly driven by a term proportional to *d*. Considering solely the undulation related energy gain, *W*_area_ results in underestimated probabilities of domain registration.

Neglecting *w* led to theoretical predictions of antiregistration [22,47]. Neglecting the spontaneous curvatures of LOD and LDD monolayers also contributed to the predictions. That is, a LOD patch in the first monolayer that is not matched by a LOD patch in the second monolayer gives rise to significant membrane bending at its edges. Restraining the membrane to a flat geometry by imposing elastic lipid deformations is energetically costly [43].

The situation is different for larger domains (*d* = 120 nm): *w_cis_* = 73.2 *k_B_T*, w_trans_ = 25.2 *k_B_T*, and *W*_area_ = 144 *k_B_T*. That is, for trans–PhoDAG–1 containing domains we see a transition in the driving force: now undulations make the major contribution to coupling. The criterion for the transition can be calculated by requiring that *γ*-and undulation-driven energies be equal to each other. This is the case for the critical diameter d* = 4Δ*γ*/*w*_area_. For *cis*-PhoDAG-1 and *trans*-PhoDAG-1 we find d*_cis_ = 60 nm and d*_trans_ = 20 nm, respectively.

Our work provides a framework for understanding how registration of nanodomains (rafts) in cell membranes may arise: By observing domains that are too small to be in register according to midplane coupling [20,22], i.e., thermal undulations, we confirm the critical role of *γ* in their genesis. Moreover, we transform nanometer sized domains from an elusive object into an optically observable entity.

## Supplementary Material

Supplementary material

## Figures and Tables

**Fig. 1:**
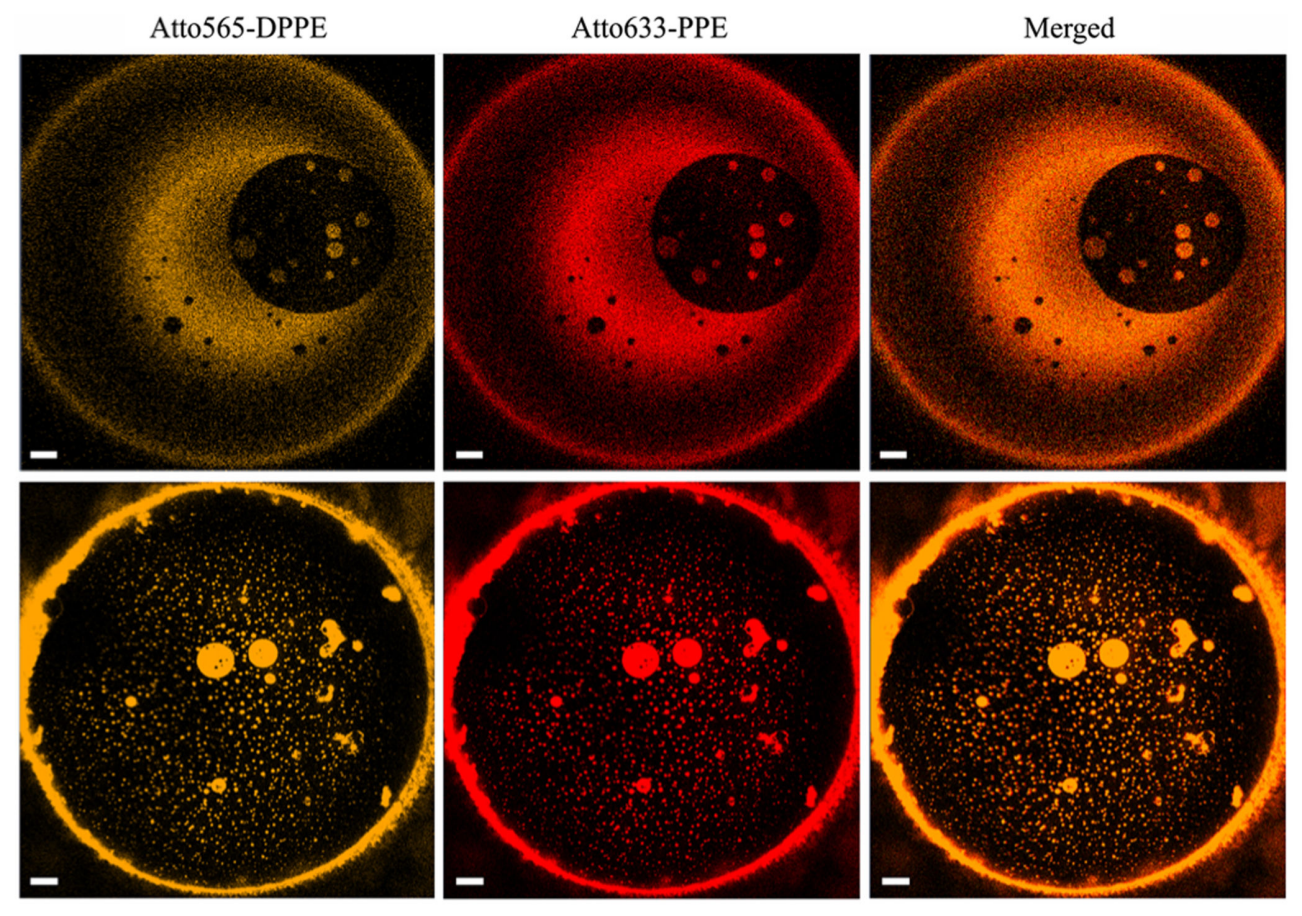
Domains of all sizes from the two membrane leafs are in register. The bright spots from the two monolayers always coincide: the left column displays micrographs that were obtained by exciting Atto565–DPPE in the *cis* monolayer; the middle column shows the fluorescence of Atto633–PPE in the *trans* monolayer of the same membrane at the same time; the right column shows perfect overlap of both channels. The upper and lower rows were obtained in two subsequent experiments at room temperature (*T* = 295 K). The lipid composition was diphytanoyl phosphatidylcholine (DPhPC): dipalmitoyl phosphatidyl choline (DPPC): photoswitchable diacylglycerol (PhoDAG–1): cholesterol 2:1:1:2 plus 0.004 mol% Atto565–DPPE in the cis monolayer and 0.004 mol% of Atto633-PPE in the *trans* monolayer. The buffer contained 20 mM HEPES and 20 mM KCl (pH = 7.0). The scale bar has a length of 20 μm.

**Fig. 2:**
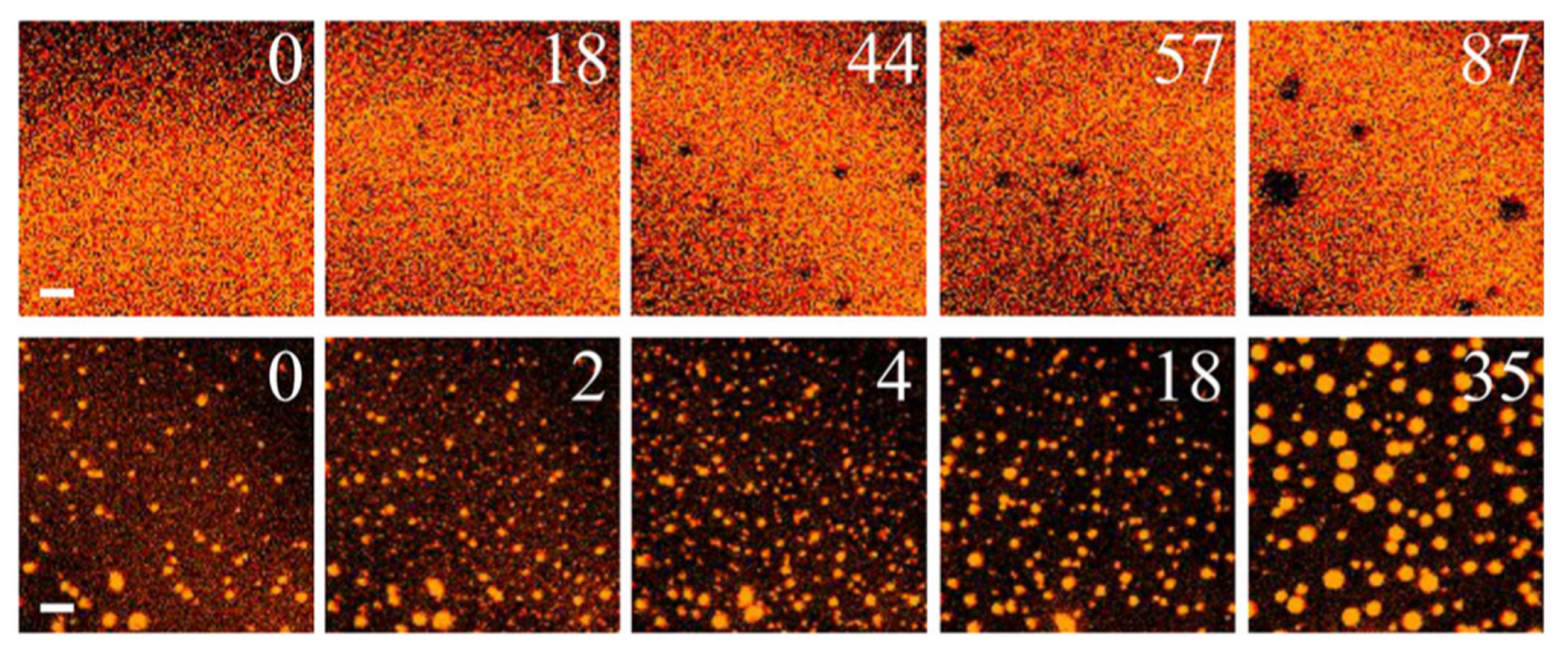
Photoinduced appearance of LODs and LDDs. Illuminating PhoDAG–1 at 460 nm via a xenon lamp coupled to a monochromator (Polychrome V, TILL Photonics GmbH, Germany) switched the lipid into its *trans* configuration. Dimmer LODs appeared that were surrounded by bright LDDs (upper row). Back switching of PhoDAG–1 into its cis configuration by exposure to light at a wavelength of 365 nm resulted in the appearance of bright LDDs within dimmer LODs. The number in the upper right corner of each frame indicates the time (in seconds) that has elapsed from the moment of photoswitching. The panels represent a superposition (merger) of the Atto565–DPPE and Atto633–PPE channels. For other conditions see Fig. 1. The scale bar has a length of 5 μm.

**Fig. 3:**
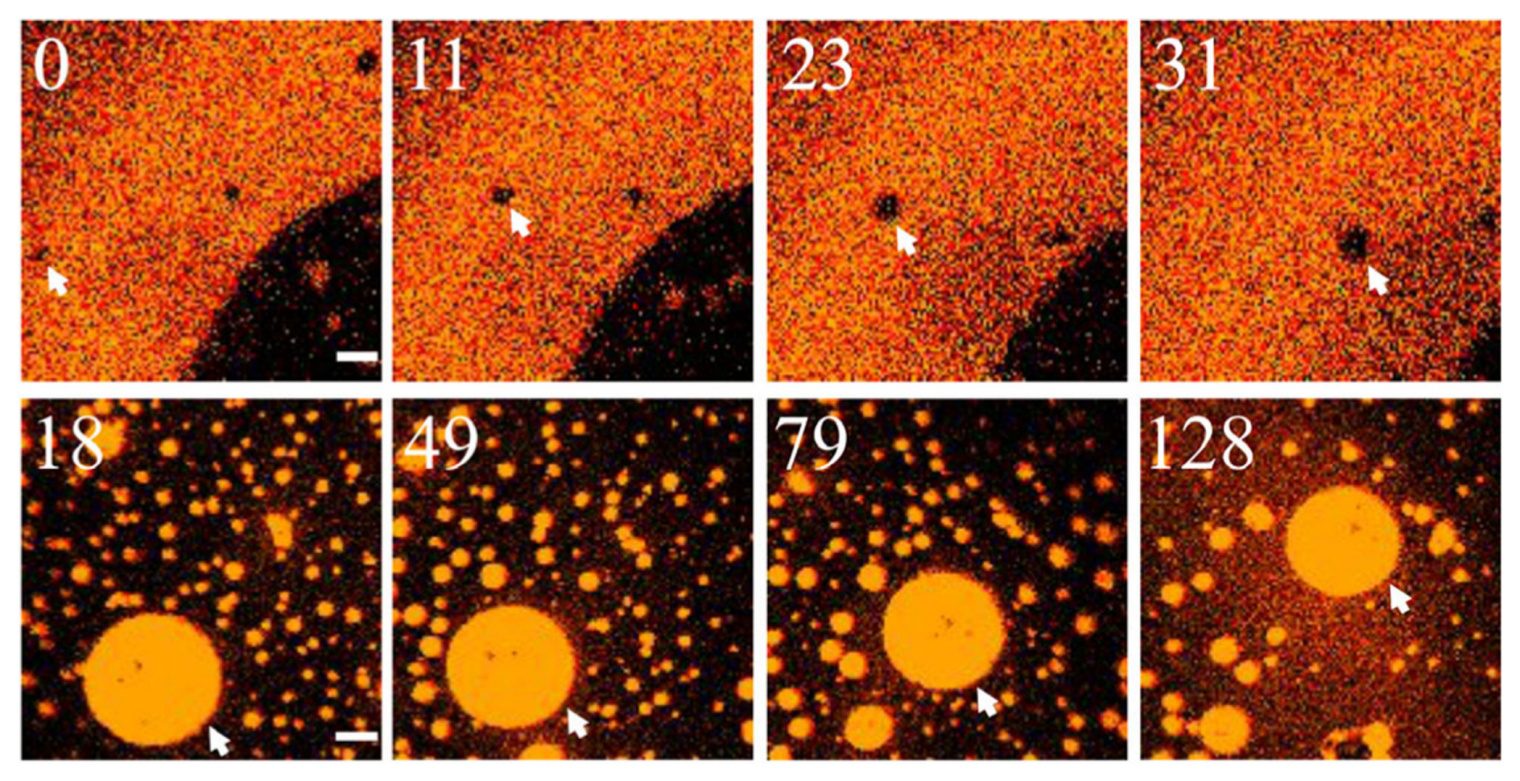
Domain size is dynamic. The growth of a LOD from *d_a_* ≈ 1.5 to *d_a_* ≈ 4.5 *μ*m occurred without merger with other domains (upper row, white arrows). Shrinkage of an LDD from an initial *d_a_* ≈ 14 to *d_a_* ≈ 11 *μ*m (white arrows, lower row) took place without visible domain patches pinching-off. The numbers in the upper left corner of each frame indicate the time (in seconds) that has elapsed after the photoswitch has been initiated. The panels represent a superposition (merger) of the Atto565–DPPE and Atto633–PPE channels. Experimental conditions were as in Fig. 1 The scale bars have a length of 5 *μ*m.

**Fig. 4:**
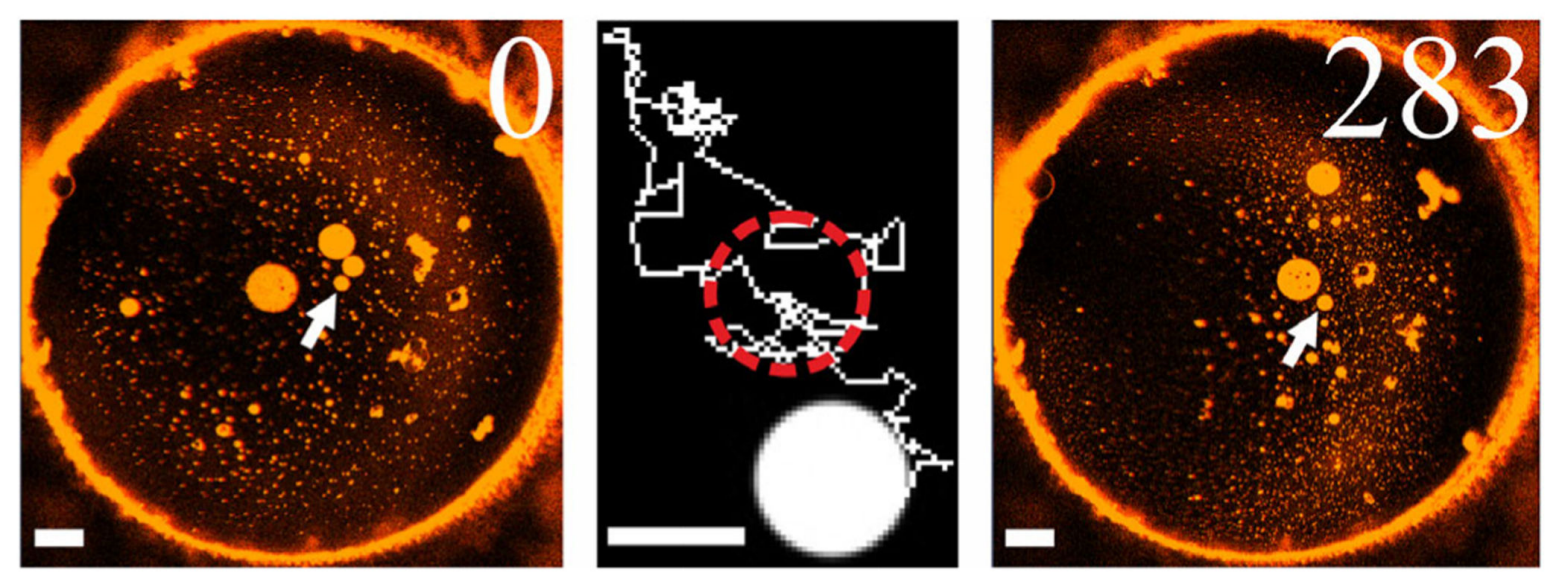
Domain tracing by confocal laser scanning microscopy. A representative trajectory (domain, see arrow) is placed between the first and the last images. The time (in s) is indicated in the upper right corner. The scale bars of the images and the trajectory are 20 and 5 *μm* in length, respectively.

**Fig. 5:**
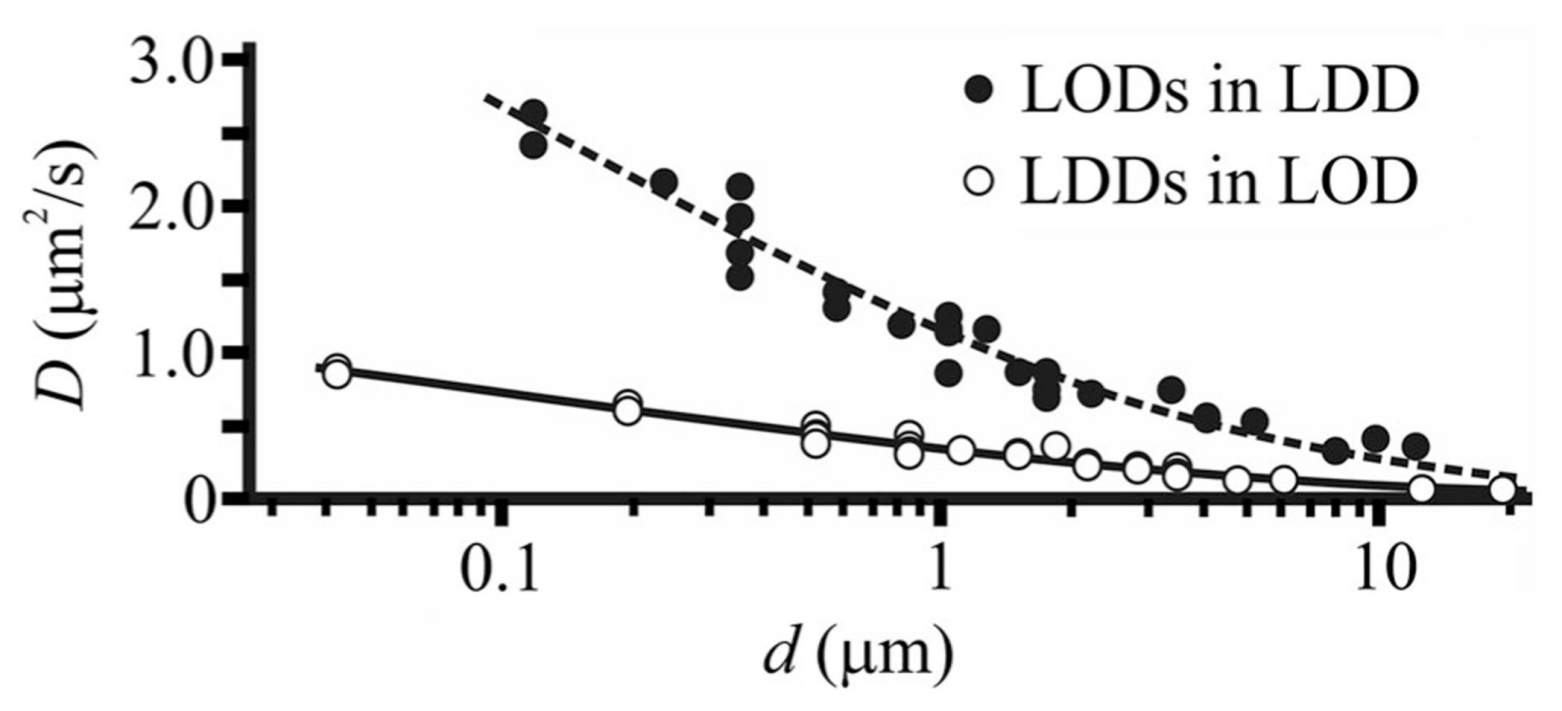
Size dependence of (i) LOD mobility in LDDs and (ii) LDD mobility in LODs. The symbols indicate experimental data, the lines represent the fits of Eq. (2) to the data. The lipid composition was (i) DPhPC:DPPC: PhoDAG–1:cholesterol 2:1:1:2 plus 0.004 mol% Atto565–DPPE in the *cis* monolayer and DPhPC:DPPC: cholesterol 2:2:2 plus 0.004 mol% of Atto633-PPE in the *trans* monolayer (filled circle), and (ii) DPhPC:DPPC:PhoDAG–1: cholesterol 2:1:1:2 plus 0.004 mol% Atto565–DPPE in the *cis* monolayer and DPhPC: DPPC:PhoDAG–1:cholesterol 2:1:1:2 plus 0.004 mol% of Atto633-PPE in the *trans* monolayer (circle).

**Fig. 6:**
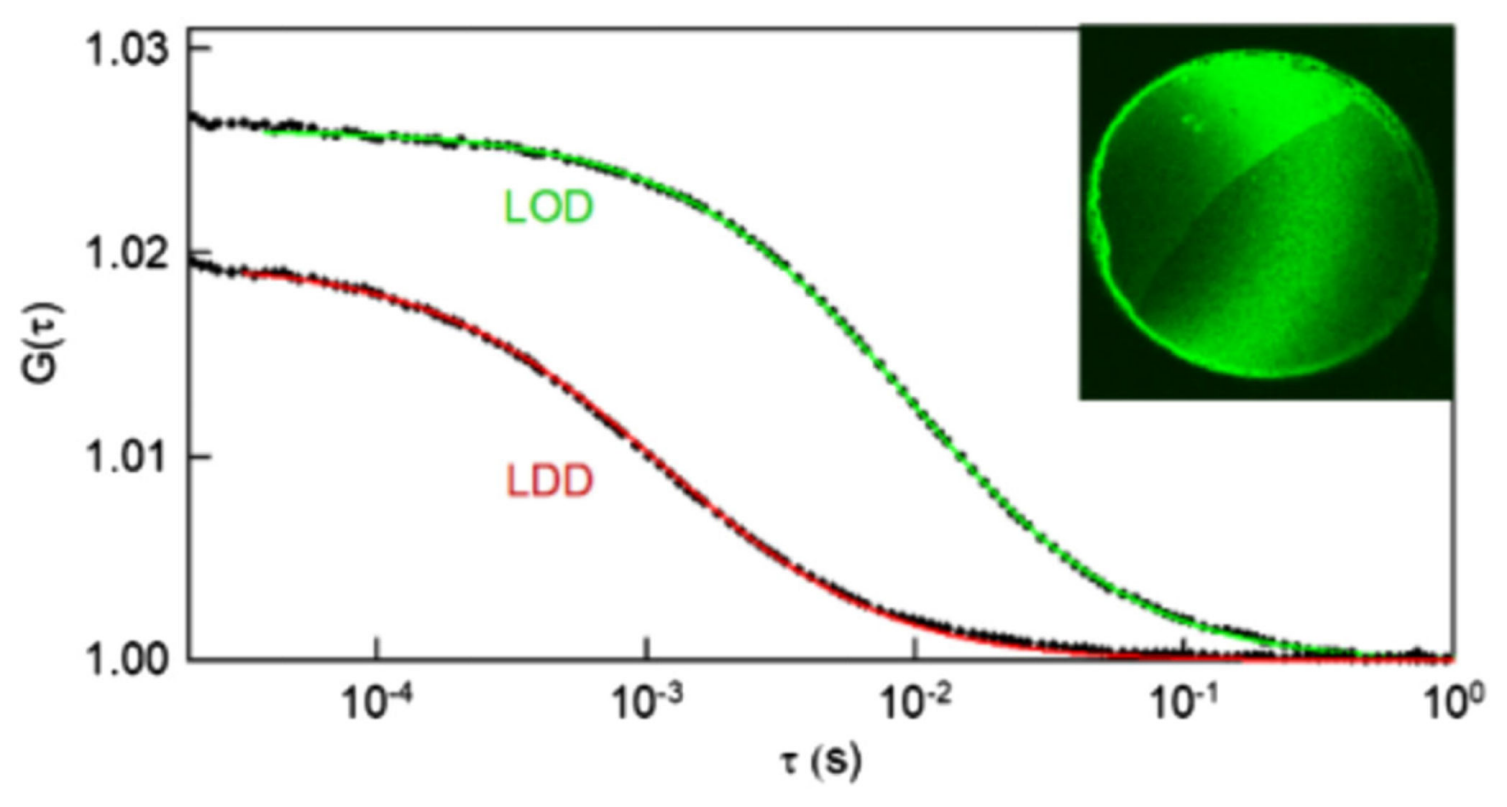
The mobility of lipid molecules in LDDs and LODs measured by fluorescence correlation spectroscopy. Dipalmitoyl-phosphatidyl-ethanolamine anchored ATTO–488 served as the probe. Representative autocorrelation functions of measured fluorescence intensities are shown as a function of time *τ* in logarithmic scale. *D* derived from fitting a two-dimensional diffusion model to the autocorrelation function for the lipid probe in LDDs and LODs agreed reasonably well with values that were predicted based on the data in Fig. 5. The inset shows an image of a planar bilayer that has been subjected to fluorescence correlation spectroscopy. The LDD is bright, while the LOD exhibits a dimmer fluorescence. The membrane consisted of one-third of DPhPC, one-third of cholesterol, and one-third of DPPC.

**Table I:** The parameters A, *β*, and *δ* of the approximation for the dependency of *D* on *d_a_* in accordance with the generalized Saffman-Delbruck relation, Eq. (2), with introduced domain diameter offset δ, Eq. (1)

Diffusing entity	A, *μ*m^2^/s	*β*	*δ, μ*m
LODs	0.76 ± 0.19	0.33 ± 0.15	0.57 ± 0.06
LDDs	0.18 ± 0.03	0.20 ± 0.06	0.46 ± 0.02

**Table II:** Spontaneous curvatures (in nm^−1^) of LOD and LDD monolayers for *cis* and *trans* configurations of PhoDAG-1

	*cis*-PhoDAG-1	*trans*-PhoDAG-1
LOD	−0.21011	−0.26795
LDD	−0.3948	−0.2414
