# Supplementary material for: Ordered Lipid Domains Assemble via Concerted Recruitment of Constituents from Both Membrane Leaflets
Source: Phys Rev Lett. Author manuscript; Available in PMC 2020 Sep 1. (PMC7115998; doi:10.1103/PhysRevLett.124.108102)
Supplement: Supplementary material [file EMS94293-supplement-Supplementary_material.pdf]

Supplementary Material for

"ORDERED LIPID DOMAINS ASSEMBLE VIA CONCERTED RECRUITMENT OF CONSTITUENTS FROM BOTH MEMBRANE LEAFLETS"

Ali Saitov[1], Sergey A. Akimov[2], Timur R. Galimzyanov[2], Toma Glasnov[3], and Peter Pohl[1]

[1]Institute of Biophysics, Johannes Kepler University Linz, Gruberstraße 40, Linz 4020, Austria
[2]A.N. Frumkin Institute of Physical Chemistry and Electrochemistry, Russian Academy of Sciences, 31/5 Leninskiy prospekt, Moscow 119071, Russia
[3]Institute of Chemistry, University of Graz, Graz, Austria

1. Apparent domain diameter d_a and true domain diameter d differ by a size independent term δ

δ has two constituents:

$$\delta = \delta_s + \delta_d, \qquad \text{S1}$$

where δ_d and δ_s are the apparent increments in d due to diffraction limitations and domain diffusion, respectively. Both δ_d and δ_s do not depend on d in a wide range of domain diameters, as we show below, thereby justifying the assumption that δ varies negligibly, i.e. $d = d_a - \delta$ for all domains.

1.1 The invariance of δ_s

Domain displacement during the time t_d of its image acquisition gives rise to a seemingly larger domain size. The effect scales with the diffusion coefficient D of the domain:

$$\delta_s \approx \sqrt{4Dt_d} \qquad \text{S2}$$

t_d depends on the apparent domain diameter d_a, more precisely on the number of lines N_L that the microscope has to scan in order to acquire the domain image. For the diameter of a single pixel d_p (~220 nm) we find:

$$N_L \approx \frac{d_a}{d_p}. \qquad \text{S3}$$

Since acquisition of 1024 lines takes about 1.7 seconds, we may write for t_d:

$$t_d \approx \frac{d_a}{d_p}\frac{1.7\text{s}}{1024} \approx c_1 d_a, \qquad \text{S4}$$

where c_1= 0.0075 s µm^{-1}. The lateral displacement of the diffusing domain during acquisition of its image can be estimated as:

$$\delta_s \approx \sqrt{4Dc_1 d_a}, \qquad \text{S5}$$

where the experimentally determined product Dd_a for d_a < 1.7 µm is equal to (i) 1.32 µm$^{3/2}$ s$^{-1/2}$ for LODs in LDDs and (ii) 0.67 µm$^{3/2}$ s$^{-1/2}$ for LDDs in LODs (SFig. 1). Accordingly, Eq. S5 returns for the respective values of δ_s: 0.22 µm and 0.12 µm.

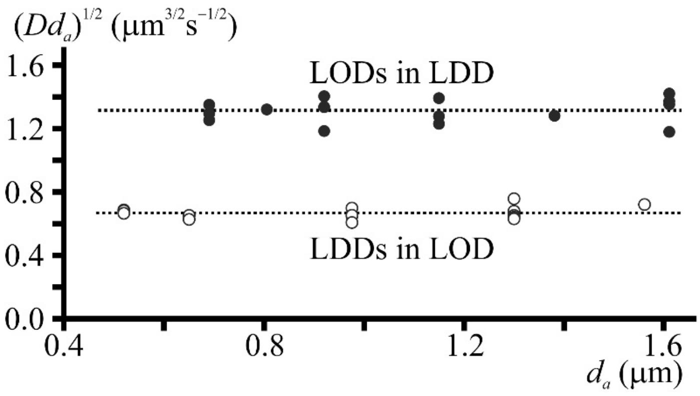

Supplementary Figure 1. The difference between apparent domain diameter (d_a) and true domain diameter (d) due to non-instantaneous image acquisition is independent of domain size. We measure (i) a smaller diffusion coefficient D and (ii) a longer scanning time (proportional to d_a) for larger domains. As a result, $\sqrt{Dd_a}$ does not depend on d_a. This is true both for LODs diffusing in LDDs (black circles) and LDDs diffusing in LODs (white circles). The horizontal dotted lines correspond to the best fit of the dependencies.

1.2 The invariance of δ_d

Diffraction limitations let domain diameter d appear larger by δ_d. δ_d does not depend on d. Rather, it is a function of both pixel size d_p and diameter of the focal area. In our case, the latter is roughly twice the size of the former (the exact ratio depending on the wavelength). Since we are taking into account only domains that are well separated in space and since every pixel contains several of fluorescent dye molecules, δ_d is equal to the distance at which fluorescence intensity (i) drops from the brightness of an LDD domain to that of an LOD domain or (ii) increases from the brightness of an LOD domain to that of an LDD domain. This distance may vary between d_p and $2d_p$ depending on the positioning of the domain boarder (red circle in SFig. 2).

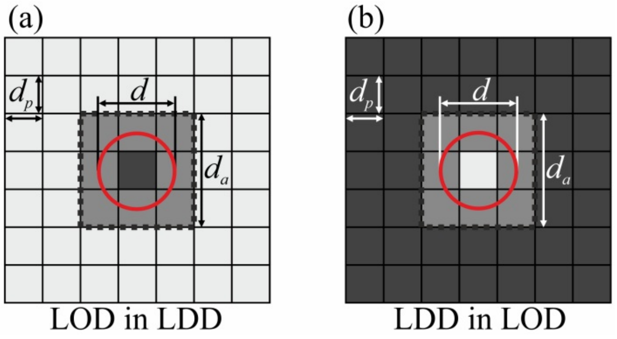

Supplementary Figure 2. Schematic representation of the diffraction induced error to the determination of domain size *d*. (a) — LOD in LDD; (b) — LDD in LOD. The domain appear to

have size d_a, because fluorescence brightness between the dimmer LOD regions (dark gray) and brighter LDD regions (light gray) does not change instantaneously, but at least over the distance d_p of one pixel (gray).

2. Selection of domain trajectories

In order to decide whether traces of single domain diffusion should enter the analysis, we calculated the straightness of the diffusion trajectory, *S*:

$$S = \frac{\left|\mathbf{x}_{N-1} - \mathbf{x}_0\right|}{\sum_{i=1}^{N-1}\left|\mathbf{x}_i - \mathbf{x}_{i-1}\right|},$$

where *N* is a number of consecutive two dimensional positions of a tracked particle (domain), $\mathbf{x}_i$ is the radius-vector of the particle center of mass at the *i*-th step of the trajectory [32]. *S* is a build in criteria of the software (Image J). We required $S \leq 0.2$. Its use for the exclusion of events that are not compatible with simple diffusion is appealing, because *S* also excludes events with rather short trajectories.

3. Domain mobility function of apparent domain radius

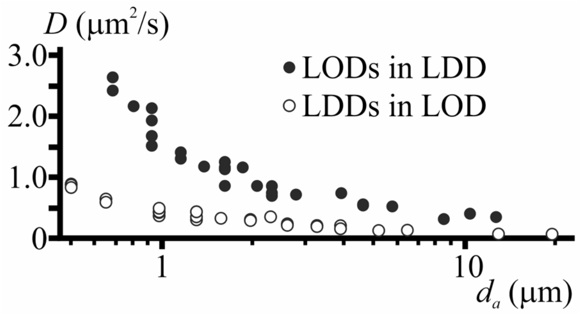

Supplementary Figure 3. Dependence of (i) LOD mobility in LDDs and (ii) LDD mobility in LODs on the apparent diameter of the domain. LOD are indicated by filled circles, LDD by empty circles. For experimental conditions see Fig. 5.
